# Polygenic risk associated with post-traumatic stress disorder onset and severity

**DOI:** 10.1038/s41398-019-0497-3

**Published:** 2019-06-07

**Authors:** Burook Misganaw, Guia Guffanti, Adriana Lori, Duna Abu-Amara, Janine D. Flory, SBPBC, Rasha Hammamieh, Aarti Gautam, Ruoting Yang, Bernie J. Daigle, Leroy Hood, Kai Wang, Inyoul Lee, Synthia H. Mellon, Owen M. Wolkowitz, Susanne Mueller, Rachel Yehuda, Marti Jett, Charles R. Marmar, Kerry J. Ressler, Francis J. Doyle

**Affiliations:** 1 Harvard John A. Paulson School of Engineering and Applied Sciences, Harvard University, Cambridge, MA USA; 2 Department of Psychiatry, Harvard Medical School and McLean Hospital, Belmont, MA USA; 3 Department of Psychiatry and Behavioral Sciences, Emory University School of Medicine, Atlanta, GA USA; 4 Steven and Alexandra Cohen Veterans Center for the Study of Posttraumatic Stress and Traumatic Brain Injury; and Department of Psychiatry, NYU School of Medicine, New York, NY USA; 5 Department of Psychiatry, James J. Peters Veterans Affairs Medical Center, Bronx, NY USA; 6 The Department of Psychiatry, Icahn School of Medicine at Mount Sinai, New York, NY USA; 7 Integrative Systems Biology, United States Army Medical Research and Material Command, United States Army Center for Environmental Health Research, Frederick, MD USA; 8 Advanced Biomedical Computing Center, Frederick National Laboratory for Cancer Research, Frederick, MD USA; 9 Departments of Biological Sciences and Computer Science, The University of Memphis, Memphis, TN USA; 10 Institute for Systems Biology, Seattle, WA USA; 11 Department of Obstetrics, Gynecology & Reproductive Sciences, University of California, San Francisco, CA USA; 12 Department of Psychiatry, University of California, San Francisco, CA USA; 13 Center for Imaging of Neurodegenerative Diseases, San Francisco Veterans Affairs Medical Center, San Francisco, CA USA; 14 Department of Radiology and Biomedical Imaging, University of California, San Francisco, San Francisco, CA USA

**Keywords:** Diagnostic markers, Clinical genetics, Psychiatric disorders

## Abstract

Post-traumatic stress disorder (PTSD) is a psychiatric illness with a highly polygenic architecture without large effect-size common single-nucleotide polymorphisms (SNPs). Thus, to capture a substantial portion of the genetic contribution, effects from many variants need to be aggregated. We investigated various aspects of one such approach that has been successfully applied to many traits, polygenic risk score (PRS) for PTSD. Theoretical analyses indicate the potential prediction ability of PRS. We used the latest summary statistics from the largest published genome-wide association study (GWAS) conducted by Psychiatric Genomics Consortium for PTSD (PGC-PTSD). We found that the PRS constructed for a cohort comprising veterans of recent wars (*n* = 244) explains a considerable proportion of PTSD onset (Nagelkerke *R*^2^ = 4.68%, *P* = 0.003) and severity (*R*^2^ = 4.35%, *P* = 0.0008) variances. However, the performance on an African ancestry sub-cohort was minimal. A PRS constructed with schizophrenia GWAS also explained a significant fraction of PTSD diagnosis variance (Nagelkerke *R*^2^ = 2.96%, *P* = 0.0175), confirming previously reported genetic correlation between the two psychiatric ailments. Overall, these findings demonstrate the important role polygenic analyses of PTSD will play in risk prediction models as well as in elucidating the biology of the disorder.

## Introduction

Post-traumatic stress disorder (PTSD) is a debilitating mental illness that can develop following a traumatic experience, such as combat, sexual assault, or natural disaster^1^. It occurs in ~10% of those experiencing severe trauma, with a lifetime incidence rate of 6.8−8% in the US general public^2,3^ and up to 15% among Operation Enduring Freedom and Operation Iraqi Freedom (OEF/OIF) veterans^4,5^. The current approach to diagnosis in general clinical practice relies on clinician interviews and patient self-reports. Variation in patients’ willingness to self-disclose, as well as highly heterogeneous symptom presentations and severity levels of PTSD^6^, make accurate and timely diagnosis challenging. Underdiagnoses, in particular, may result in serious, and at times fatal, outcomes that could have potentially been avoidable^7–10^.

The urgent need for biomarkers as an objective diagnostic and prognostic tool for PTSD cannot be overstated^11,12^. Despite an international effort studying military and civilian cohorts where many molecular layers and modalities were investigated^13–16^, there are, as of yet, no validated blood-based PTSD biomarker panels. Towards this end, one of the more-promising approaches, facilitated by a recent large-scale multi-site collaborative genome-wide association study (GWAS) from the Psychiatric Genomics Consortium for PTSD (PGC-PTSD)^17^, is genomic profiling using single-nucleotide polymorphisms (SNP’s).

PTSD genomic profiles assess the degree of genetic propensity, in probabilistic terms, for developing PTSD following a traumatic experience. This information is of great importance not only for identifying biomarkers for disease prognosis, but also for elucidating disease etiology and mechanisms. As genetic profiles can be obtained prior to trauma exposure, they can also be used to plan preventative measures in at-risk populations, including military personnel. For example, duty assignments, number of tours, and dwell times between tours can be adjusted in relation to risk and resilience profiles. Pre-deployment resilience building strategies and personalized early interventions can also be implemented, especially for those that are at a higher risk. Furthermore, in the long-term, enhanced understanding of the genetics of the disorder will inform the design and tailoring of effective therapeutics.

The main technical challenge in building genomic profiles, besides shortage of study samples, is the fact that the genetic architecture of PTSD, not unlike most other complex psychiatric traits^18^, is highly polygenic. Individual (or even a few dozen) common SNP variants account for only a small part of the genetic influence. For instance, the largest published PTSD GWAS to date^17^, done with 20,000 (25% cases) participants, could not find any novel GWAS significant variant, nor could it replicate previously identified hits. A study of this sample size had 80% power to detect a disease (causative) allele with genotype relative risk of 1.186–1.35 (assuming an additive model with disease allele frequency of 5–20% and a prevalence of 8% requiring a significance level of 5e-8). This suggests that common variants have individually small effect-sizes and are not by themselves predictive of PTSD risk.

Despite this lack of individual large effect-size common variants, small effects from many variants accumulate to result in a moderate level of heritability. Among those exposed to trauma, twin studies indicated a PTSD heritability of ~30% in men and 70% in women^19,20^. Also of note, a moderate level of heritability (30%), particularly for women, was recently confirmed with SNP array-based heritability analysis^17^. Hence, a sensible way of capturing the genetic liability of an individual is, instead of looking at individual genes and variants in isolation, to account for the additive effects of these small effect risk variants. The total sum of risk variants, weighted by corresponding effect-sizes, which are usually obtained from GWAS summary statistics, is commonly known as polygenic risk score (PRS)^21,22^.

We investigated various issues pertaining to PTSD-PRS. First, we discussed its opportunities and limitations from a theoretical performance analysis. Next, we constructed the PRS using GWAS summary statistics in a deeply phenotyped and well-curated cohort comprised of OIF/OEF veterans conducted by Systems Biology PTSD Biomarkers Consortium (SBPBC), hereafter referred to as the SysBio cohort. We then showed that ancestral makeup similarity between discovery and validation cohorts was a major performance determinant. Furthermore, as a demonstration of genetic overlap among psychiatric illnesses, we use schizophrenia GWAS summary statistics to predict PTSD phenotypes. Overall, in addition to theoretical and empirical investigation of PRS prediction performance on PTSD onset and severity, we demonstrated its use in studying genetic correlation with other psychiatric disorders.

## Methods

### Participants

Study participants are OEF/OIF veterans recruited from New York University Langone Medical Center (NYU), the James J Peters VA Medical Center (JJPVAMC), and Icahn School of Medicine at Mount Sinai (ISMMS) as part of a multi-site consortium effort (SBPBC) to identify, validate, and deploy PTSD diagnostic biomarkers. All participants in both cases and controls had experienced combat exposure. Written informed consent was obtained from all participants before the clinical assessment was conducted. Assessment of combat-associated PTSD diagnosis and severity was based on CAPS-IV (Clinician Administered PTSD Scale for DSM-IV) administered by a doctoral-level clinician. Deep and extensive phenotype information was thoroughly gathered. These included, in addition to CAPS, the Structured Clinical Interview for DSM-IV for anxiety, mood, alcohol and substance use, and psychotic disorders, as well as demographics data including race/ethnicity, age, relationship-status, and anthropometric data, including BMI, weight, and height. Further details about clinical and demographic data can be found in Table 1. In order to maximize signal detection, those with intermediate severity level sub-threshold PTSD were excluded. Other exclusion criteria included (i) any drug abuse within a year of assessment, (ii) lifetime schizophrenia, bipolar disorder, obsessive-compulsive disorder or other psychotic disorders, (iii) head injury with current post-concussion symptoms, (iv) trauma exposure within 3 months of assessment to exclude non-combat-associated PTSD, and (v) current suicidal or homicidal ideation. Of the genotyped subset, 116 participants are PTSD cases (CAPS range: 37–102, median: 65.5), whereas the other 128 are trauma exposed, age and ethnicity matched, healthy controls (CAPS range: 0–24, median: 2).

**Table 1:** Sample characteristics by PTSD status of SysBio cohort included in this study

	PTSD cases (*n* = 128)	Healthy controls (*n* = 116)	*P* value
CAPS cur	2.00 (0.00, 6.00)	65.50 (51.75, 80.25)	<0.001
CAPS LT	8.00 (3.00, 15.00)	90.00 (77.75, 101.00)	<0.001
Female	14% (18)	14% (16)	0.952
BMI	27.35 (24.45, 29.85)	28.25 (25.61, 32.28)	0.028
Age	30.00 (27.75, 37.00)	31.00 (28.75, 36.25)	0.338
*Race/ethnicity*			
Asian	7% (9)	3% (3)	
Black	23% (30)	29% (34)	
Hispanic	28% (36)	42% (49)	0.026
White	38% (49)	24% (28)	
Other	3% (4)	2% (2)	
*Education*			
1	2% (3)	3% (4)	
2	20% (26)	35% (41)	
3	24% (31)	30% (35)	0.009
4	35% (45)	25% (29)	
5	17% (22)	6% (7)	
6	1% (1)	0% (0)	
BDI	3.00 (0.00, 9.25)	24.00 (16.50, 31.00)	<0.001

CAPS, Clinician Administered PTSD Scale (cur: current and LT: Life-Time); BMI, Body Mass Index; BDI, Beck Depression Inventory II total score (*n* = 239)

For continuous variables, Q2 (Q1, Q3) represent the median, the lower quartile, and the upper quartile, respectively. For categorical variables, percentages (and frequencies) are shown. Wilcoxon rank sum test for continuous variables and Pearson *χ*^2^ test for categorical variables are used. Education levels: 1, Up to 12th grade; 2, H.S. Diploma or GED; 3, 2 yrs. college A.A. Degree; 4, 4 yrs. College Bachelor’s Degree; 5, Masters Degree; 6, Doctoral Degree

### Raw genotype data, imputation, and quality–control of target cohort

Blood samples were drawn at JJPVAMC or ISMMS and shipped to Emory University for SNP genotyping. The genotype data were obtained with the Infinium PsychArray BeadChip from Illumina (San Diego, CA, USA). Genotype calling was made with GenomeStudio. Samples were processed in two batches (owing to different sample arrival times). Per-sample genotyping rate, in each batch, was >99%, resulting in a total of 303,378 typed variants. Hence, no sample needed to be discarded due to quality–control (QC). This leaves 244 samples with genotype and phenotype data. Imputation was performed with standard steps. First, the genotype data were split into individual chromosomes. Then, strand orientation of genotyped data of each autosomal chromosome were checked and corrected with PLINK^23^. As pre-phasing was believed to improve imputation accuracy and speed, the study data were pre-phased with SHAPEIT^24^ with genetic map data for build 37^25^. Imputation was done for a window of 5 Mb at a time with IMPUTE2^26^ using phased reference panel from 1000 Genome Project phase 3 data set. The imputed data were reassembled with GTOOL. The following criteria were used for QC filtering with PLINK: minimum threshold for a minor allele frequency of 0.01, maximum individual missingness rate of 0.1, and Hardy–Weinberg equilibrium *p*-value of 0.001. A total of 9,831,409 variants survived this QC filtering step. Of note, our target SysBio cohort participants were completely independent of the discovery GWAS cohort of the PGC-PTSD study.

### GWAS summary statistics data

PRS is often trained on GWAS summary statistics data, without the need to directly access raw individual-level genotype training data, which is often not readily available. Reliable estimation of PRS parameters, however, requires a large sample size GWAS. GWAS summary statistics data, unlike individual-level genetic data, are often publicly available for many traits and diseases. It typically contains results from univariate association test statistics on a variant-per-row format. In this study, GWAS summary statistics data from two studies were used as base/discovery data sets:^1^ PTSD GWAS summary statistics data, which is the largest published PTSD study to date (*n* = 10 *k*, 25% cases) with European ancestry participants^17^, and^2^ Schizophrenia GWAS summary statistics data, which is the largest psychiatric genetic study (37 K cases and 113 K controls) to date, also consisting of mostly European ancestry participants^27^.

### LD clumping, *P* value thresholding, and computing PRS

To choose the optimal predictive set of SNP’s on the target data set, we conducted the standard LD clumping followed by *P* value thresholding procedure. The LD clumping was done on windows of 250 kb with a squared correlation of allele counts *r*^2^ = 0.1. This means that within a given 250 kb window with *r*^2^ = 0.1, the SNP with the smallest *p* value was chosen as a representative SNP. A PTSD-PRS was constructed and its performance (Nagelkerke *R*^2^, a measure of coefficient of determination for binary traits) was evaluated over a grid of ten equally spaced *p* value thresholds from 0.1 to 1 inclusive, and the nominal significance threshold of 0.05 (Fig. S1). Each time only SNP’s with lower *P* value than the threshold were included in the PRS summation and the *p* value threshold with the best performance (*P*_*T*_ = 0.2) was chosen for the final PRS calculation. For schizophrenia–PRS, a threshold of 0.05 had been shown to be the most predictive in the original publication^27^. Thus, this threshold was used to avoid multiple testing burden. The first five principal components were added as covariates to correct for population stratification in both analyses. Standardization was done by converting raw scores to *z* scores (centering by mean and scaling by standard deviation). This part of the analyses was done with a wrapper function around the R code of PRSice v1.25^28^.

### PRS

PRS summarizes genetic liability from many variants into a single number as a weighted sum of per-loci risk allele dosage^21^. More precisely, ${\mathrm{PRS}}_{tj} = \mathop {\sum }\nolimits_{{\mathrm{i}} \in {\mathrm{S}}} \hat \beta _{ti}x_{ij},$ where $x_{ij} \in \left\{ {0,1,2} \right\}$ is the additively coded allele frequency of the ith marker for the jth individual, *S* is a set of SNP’s that survived the clumping and thresholding steps, *t* is one of the two traits studied as base phenotypes, and $\hat \beta _{ti}$ is estimated effect-size (log odds ratio or regression coefficient) obtained from GWAS summary statistics on the base phenotype, which may be genetically correlated, but not necessarily the same as the target phenotype. In our case, the base phenotype is either PTSD or schizophrenia, depending on the discovery data set used in the analyses (PGC-PTSD or PGC-schizophrenia), whereas the target phenotype is PTSD diagnosis.

### Genetic clustering to evaluate ancestry

The clustering was performed with PLINK^23^. First variants were filtered with the QC criteria described above. The resulting set of SNP’s was LD (linkage disequilibrium) pruned with window size of 50, shift size of 5 and correction (*r*^2^) threshold of 0.2 (–indep-pairwise 50 5 0.2). Then IBS (identity-by-state) similarity between individuals was computed (–genome) with the pruned data. Clustering was performed with this similarity matrix (–cluster). This yielded the four clusters shown in Fig. S2. Self-identified ethnicity/race composition of the clusters are shown in Fig. S2. Cluster 1 primarily contains Asians and some Hispanics. Cluster 2 contains almost all Whites and some Hispanics. Cluster 3 contains almost all Blacks. Cluster 4 mostly consists of Hispanics (See Table S1 for details).

### Additional statistical analyses

*R*^2^ of the PRS was computed as the difference between the *R*^2^ of full model that contains PRS along with covariates and the *R*^2^ of null model that contains only covariates. Similarly, Nagelkerke’s pseudo-*R*^2^ of the PRS was computed as the difference in Nagelkerke’s pseudo-*R*^2^ between that of the full model and null model. The *p* value of the null hypothesis that the regression coefficient of PRS is zero is reported with the *R*^2^. Odds ratios and difference in mean CAPS (and corresponding 95% confidence intervals) between quantiles were computed using the glm function in R (with family = “binomial” or family = “gaussian”), with the first five principal components added as covariates to control for population stratification. The power analysis for single variants in the Introduction section (for the previous largest GWAS) was done using GAS power calculator. Standardization was done by converting raw scores to *z* scores (centering by mean and scaling by standard deviation). All computations were done on R (version 3.2.3) statistical computing environment and PLINK (v1.90b3s 64-bit)^23^.

## Results

### Projections from theoretical analysis

**Fig. 1:**
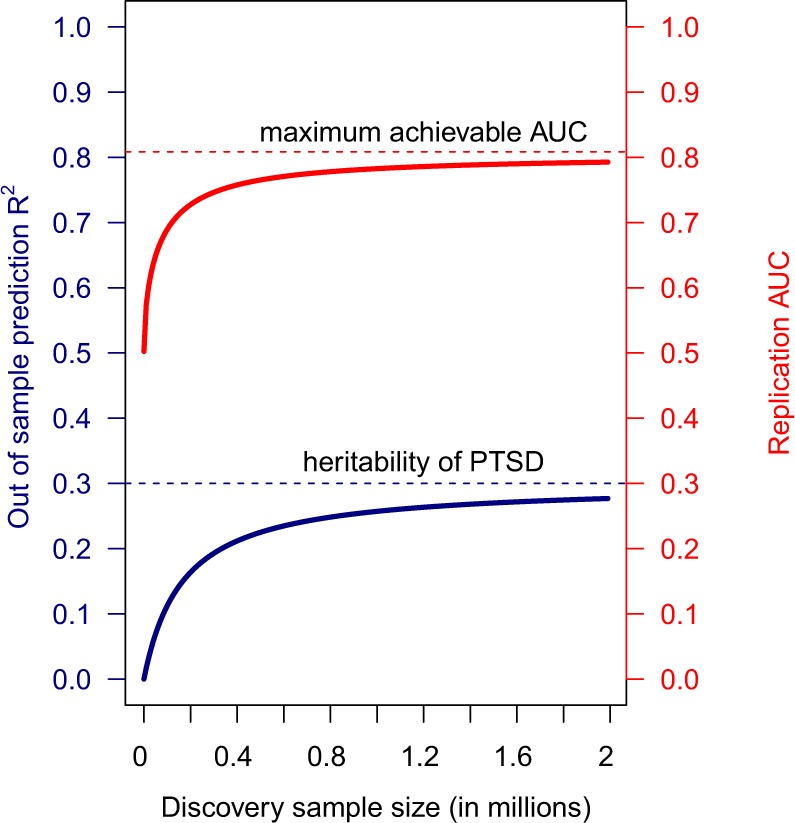
Performance projections and upper bound of a genomic predictor for PTSD onset. Assuming 50,000 non-null contributing markers, coefficient of determination (fraction of variance explained) and corresponding AUC’s of genomic profiles built on finite number of samples are plotted in blue and red, respectively

Initially, we sought to provide a preview of the roadmap ahead using analytical derivation. In light of upcoming large-scale genetic studies, this approach will also set expectations for opportunities and limitations for future genetic risk prediction studies of PTSD. These projections are predicated on standard assumptions and models from quantitative genetic theory (Supplementary Materials). Using the heritability estimate of 30% (obtained from early male twin studies and recent SNP heritability estimates for women) and an estimated disease prevalence of 8%, the optimal panel trained on an infinite number of samples would have an AUC of a little over 80% (Fig. 1). It should be noted that unlike most study samples, including the present study samples, where cases are intentionally oversampled so as to make up half of a study cohort (i.e., ascertainment), both the training sample (whose sample size is shown in the horizontal axis) and replication sample (whose performance is shown in the vertical axes) are assumed to be drawn randomly and independently from the general public where disease incidence rate is 8%.

### Constructing PTSD-PRS from GWAS summary statistics

We used the two GWAS summary statistics data from the largest PTSD GWAS study published to date conducted by PGC-PTSD:^17^ one performed on European ancestry cohorts and another on African ancestry. Each study consists of ~10,000 samples with ~25% PTSD positive cases. The African ancestry summary statistics did not result in any statistically significant predictive PRS, even when considering only African–American (or cluster 3) subset of our target subjects. This might arise from several technical challenges, including the fact that the African genome is highly diverse with short LD blocks and recent admixtures^29–31^, and most commercial arrays that tag a single variant from an LD block have low genetic coverage for African ancestry genome^32^. For this reason, only the European (EA)-based summary statistics are used in the present study to build PTSD-PRS for all target subjects including those of African ancestry participants.

The PRS was constructed with a clumping-and-thresholding approach with the first five principal components added as covariates (see Methods). The PRS constructed with the best performing *P* value threshold (*P*_T_ = 0.2, which accounts for 24,034 independent variants) explained ~5% of PTSD onset variance (Nagelkerke *R*^2^ = 4.68%, *P* = 0.003) as well as PTSD severity variance (*R*^2^ = 4.35%, *P* = 0.0008) as measured by CAPS (a quantitative measure of PTSD severity that ranges from 0 to 120).

**Fig. 2:**
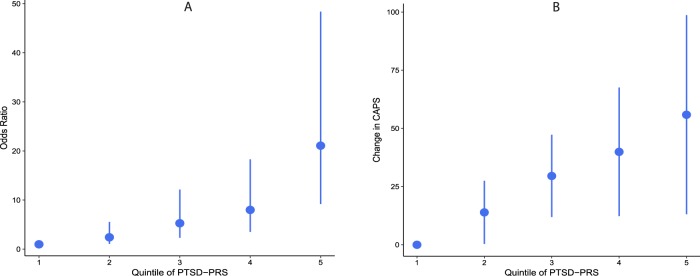
PTSD onset and PTSD severity and stratification into risk groups with PRS. The first (lowest) quintile is used as a reference. For every other quintile, the mean difference in CAPS (or the odds ratio of PTSD onset) from the first quintile is plotted (corresponding to the dot in the plot). The bars indicate 95% confidence intervals around the mean differences (or odds ratios)

For all genotyped samples pooled together (*n* = 244), the PTSD-PRS has an AUC (area under the receiver operating characteristic curve) of 0.60 for PTSD diagnosis and a C-index of 0.58 for prediction of CAPS. Next, using the PRS values, participants are stratified into equal-sized quantiles. The odds ratio for PTSD diagnosis between the highest and lowest quartiles is 11.2 (95% CI = 5.4–23.1), and between highest and lowest deciles is 50.5 (95% CI = 15.9–160) (Fig. 2). Similarly, the difference in mean CAPS between the highest and lowest quartiles is 40.7 (95% CI = 0.9–80.5), and between highest and lowest deciles is 58.3 (95% CI = −12.5–129.1) (Fig. 2).

### Ancestral composition is a major performance factor

As our cohort comprises ethnically diverse participants, reflective of the diversity among those serving in the US military, we examined the degree to which genetic ancestry affects PRS prediction performance. In order to define more genetically homogeneous subgroups of our cohort, instead of using self-identified ethnicity/race, hierarchical clustering was performed with the genetic data in an unsupervised manner (i.e., without making use of self-identified ethnicity/race label), see Methods. Having defined four genetic ancestry clusters (Fig. S2), we examined the performance of the PRS in the four clusters separately. Not surprisingly, the EA PRS has the most predictive value for clusters 1 and 2 (Table S1). In contrast, its performance on clusters 3 and 4 are near one representing a random classifier. It should be noted that limitations of PRS to predict across ancestral groups has been reported for other traits as well^33,34^.

### Cross-disorder prediction with schizophrenia–PRS

One of the most-profound findings emerging from recent psychiatric genomic studies is the degree to which psychiatric disorders, as defined and classified based on conventional diagnostic nosologies, overlap at a genetic level^35,36^. To demonstrate this point for PTSD, we chose the largest (*n* = 150 *K*) and most successful (108 significant hits) PGC study to date conducted in schizophrenia^27^, and showed evidence of polygenic overlap with PTSD. For schizophrenia–PRS, the step of choosing the optimal *p* value threshold (*P* value thresholding) is skipped in order to avoid multiple testing burden. Instead, a threshold of nominal significance (*P*_*T*_ = 0.05) has been shown to be the most predictive in the original publication^27^, and that threshold is used here.

The resulting PRS explains ~3% of the variance in predicting PTSD onset (Nagelkerke *R*^2^ of 2.96%, *p* = 0.0175). The corresponding AUC and C-index are 0.57 for both. For stratification, odds ratio for PTSD diagnosis between the highest and lowest quartiles is 4.5 (95% CI = −4.4–60.8), and between highest and lowest deciles is 12.6 (95% CI = 3.9–40.9). The difference in mean CAPS between the highest and lowest quartiles is 28.2 (95% CI = −4.4–60.8), and between the highest and lowest deciles is 53.6 (95% CI = 12.3–94.98).

## Discussion

In this study, we have demonstrated that the PRS constructed from currently published GWAS results has significant, albeit insufficient for clinical use, discrimination and stratification ability for predicting PTSD diagnosis, as well as symptom severity. Theoretical analysis indicates the remaining potential of the PRS that is yet to be realized. Furthermore, the prediction ability of schizophrenia–PRS on PTSD outcomes points to the existence of polygenic overlap between PTSD and schizophrenia, confirming previously reported genetic correlation between the two disorders.

We believe that three aspects of PRS construction merit particular attention and need to be explored further in future studies. First, the method employed to construct the PRS. Conventional machine learning approaches, where the model is trained on raw genotype data, have been reported to outperform the GWAS-based approach used here^37^. However, such approach was not feasible because raw genotype data in large-scale studies were not available. In a GWAS-based approach, summary statistics data of GWAS are used to estimate risk score coefficients of genotype dosage. After initial use in schizophrenia^21^, this approach has proven successful in capturing and predicting the genetic influence on multiple complex polygenic traits^38,39^. Here we showed a PRS constructed in a GWAS-based approach successfully stratified patients into risk groups with distinct PTSD risk and severity levels in a cohort that is independent of the discovery GWAS samples. We expect uncertainties in the likelihoods and estimates will become lower as more data are amassed. The expected rate of this improvement is estimated from a theoretical analysis. Furthermore, advances in novel methodological approaches may accelerate this pace. Most notably, recent methods leverage information on genetically related traits to improve power of univariate association statistics^40^ or to improve polygenic prediction performance^41,42^.

Second, future polygenic risk prediction models, in addition to common single-nucleotide variants studied in this article, can incorporate rare and low frequency variants^43,44^ and other complex structural polymorphisms (for example, copy number variations that have been shown to be important for psychiatric disorders^45^). Given the rapidly evolving technological developments in whole genome and exome sequencing, this is an avenue that will become possible in the very near future. Once identified, these rare variants are likely to have larger effect-sizes (negative selection), and have potential to substantially improve prediction accuracy. Integrating other modalities, including neuroimaging biomarkers and other omics panels such as epigenomics, transcriptomics, metabolomics, and proteomics, is also promising.

Third, the PRS predicted phenotype is an important factor to consider for future studies. PTSD is characterized by a heterogeneous set of distinct symptoms. PTSD-PRS, as applied in the current study, attempts to predict genetic influences on the overall diagnosis, ignoring heterogeneity in the clinical presentation. As larger genotyped samples that are more deeply phenotyped become available, it will be possible to create genetic scores for clinical subtypes (for example, dissociative and depressive subtypes) and sub-phenotypes (for example, the four symptom clusters of PTSD) as well as specific traits, some of which might be shared with other disorders. This is particularly valuable for PTSD, and psychiatric illnesses in general, where comorbidity is prevalent and the boundaries around symptom-based diagnostic criteria are a moving target. This approach may also unearth pleiotropic patterns and help explain the widespread genetic correlations among psychiatric disorders and behavioral traits.

Ethnic diversity in genetic study cohorts (as is the case for a cohort consisting of US military members or, for that matter, the nation’s population at large) presents both unique challenges and opportunities. On one hand, beyond the mere proportional representation of the diverse US military service men and women, a genetically diverse study sample facilitates identification of trans-ethnic and population-specific causal variants^46^. On the other hand, genetic predictors trained on a GWAS conducted on a given ancestral group is less predictive in samples from a different ancestral group. As most genetic studies are conducted with European ancestry participants^47^, the prediction for non-Europeans is more difficult, particularly for African ancestry individuals, as is seen in the present study.

Going forward, it is important to keep both pros and cons of genetic biomarkers in mind. One of the reasons genetic biomarkers are attractive for psychiatric traits is the fact that samples from in vivo brain tissue, the primary disorder-relevant tissue for a psychiatric illness, is usually inaccessible. Most other “-omics” markers have tissue-specific variation, with peripheral profiles not aligning with those from the brain. Also, in addition to being a more stable marker, presently available technologies for genetic markers have a better analytical validity than other omics assays. On the other hand, information content from a single-molecular layer might be inherently limited (as shown here for genetic predictors with theoretical analyses). In order to build a robust biomarker panel, combining multiple modalities might be necessary. Addressing ethical concerns and potential misuses of genetic information also should be considered^48^.

Limitations of the study need to be noted. First and foremost, the current PRS has sub-optimal predictive accuracy owing in part to the fact that the discovery GWAS is still underpowered. Our target cohort is also small and comprises very well-curated samples that is not a random representative sample from the general population. Here, we almost exclusively used data from male participants. Future studies need to include larger numbers of female participants, particularly in light of the fact that women have double the rates of PTSD heritability and prevalence. Also, preliminary findings on gender-specific mechanisms of the illness have been reported^49,50^. In addition, functional interpretation of the PRS is also difficult owing to the large number of genetic variants it comprises.

In summary, our work contributes to the use of polygenic risk for a further understanding of PTSD risk and its underlying mechanisms, whereas also identifying areas of needed future research. Overall, these findings showed that PRS, in addition to being a powerful prognostic tool, is useful in unravelling disease etiology and mechanisms, which, in turn, will enable more personalized and novel intervention strategies. As more well-powered genetic studies become available in the near future, together with advances in whole-genome and exome sequencing, accuracy, and insight obtained from such analyses will become even more precise and useful clinically.

## Supplementary information

Supplementary information - Polygenic risk associated with post-traumatic stress disorder onset and severity

## References

[1] Yehuda R (2015). Post-traumatic stress disorder. Nat. Rev. Dis. Prim..

[2] Kessler RC (2005). Lifetime prevalence and age-of-onset distributions of DSM-IV disorders in the National Comorbidity Survey Replication. Arch. Gen. Psychiatry.

[3] Vieweg WVR (2006). Posttraumatic stress disorder: clinical features, pathophysiology, and treatment. Am. J. Med..

[4] Seal KH, Bertenthal D, Miner CR, Sen S, Marmar C (2007). Bringing the war back home: mental health disorders among 103 788 US veterans returning from Iraq and Afghanistan seen at Department of Veterans Affairs Facilities. Arch. Intern. Med..

[5] Ramchand R., Karney B. R., Osilla K. C., Burns R. M., Caldarone L. B. Prevalence of PTSD, depression, and TBI among returning servicemembers. In *Invisible Wounds of War: Psychological and Cognitive Injuries, Their Consequences, and Services to Assist Recovery*. (eds. Tanielian T., Jaycox L. H.) 35–86 (RAND Corporation; 2008).

[6] Galatzer-Levy IR, Bryant RA (2013). 636,120 ways to have posttraumatic stress disorder. Perspect. Psychol. Sci..

[7] Sareen J (2007). Physical and mental comorbidity, disability, and suicidal behavior associated with posttraumatic stress disorder in a large community sample. Psychosom. Med..

[8] Hendin H, Haas AP (1991). Suicide and guilt as manifestations of PTSD in Vietnam combat veterans. Am. J. Psychiatry.

[9] Jakupcak M (2009). Posttraumatic stress disorder as a risk factor for suicidal ideation in Iraq and Afghanistan war veterans. J. Trauma Stress..

[10] Gradus JL, Suvak MK, Wisco BE, Marx BP, Resick PA (2013). Treatment of posttraumatic stress disorder reduces suicidal ideation. Depress Anxiety.

[11] Shalev A, Liberzon I, Marmar C (2017). Post-traumatic stress disorder. N. Engl. J. Med..

[12] Insel T (2010). Research Domain Criteria (RDoC): toward a new classification framework for research on mental disorders. Am. J. Psychiatry.

[13] Neylan Thomas C., Schadt Eric E., Yehuda Rachel (2014). Biomarkers for combat-related PTSD: focus on molecular networks from high-dimensional data. European Journal of Psychotraumatology.

[14] Kang HJ, Yoon S, Lyoo IK (2015). Peripheral biomarker candidates of posttraumatic stress Disorder. Exp. Neurol..

[15] Schmidt U, Kaltwasser SF, Wotjak CT (2013). Biomarkers in posttraumatic stress disorder: overview and implications for future research. Dis. Markers.

[16] Tylee DS (2015). Blood-based gene-expression biomarkers of post-traumatic stress disorder among deployed marines: a pilot study. Psychoneuroendocrinology.

[17] Duncan L. E. et al. Largest GWAS of PTSD (*N* = 20 070) yields genetic overlap with schizophrenia and sex differences in heritability. *Mol. Psychiatry.* **23**, 666–673 (2017). 10.1038/mp.2017.77 PMC5696105 28439101

[18] Geschwind DH, Flint J (2015). Genetics and genomics of psychiatric disease. Science.

[19] True WR (1993). A twin study of genetic and environmental contributions to liability for posttraumatic stress symptoms. Arch. Gen. Psychiatry.

[20] Sartor CE, McCutcheon VV, Pommer NE, Nelson EC, Grant JD, Duncan Ae (2011). Common genetic and environmental contributions to post-traumatic stress disorder and alcohol dependence in young women. Psychol. Med..

[21] Purcell SM (2009). Common polygenic variation contributes to risk of schizophrenia and bipolar disorder. Nature.

[22] Torkamani A, Wineinger NE, Topol EJ (2018). The personal and clinical utility of polygenic risk scores. Nat. Rev. Genet..

[23] Purcell S (2007). PLINK: a tool set for whole-genome association and population-based linkage analyses. Am. J. Hum. Genet..

[24] Delaneau O, Marchini J, Zagury J-F (2012). A linear complexity phasing method for thousands of genomes. Nat. Methods.

[25] Delaneau O (2014). Integrating sequence and array data to create an improved 1000 Genomes Project haplotype reference panel. Nat. Commun..

[26] Howie B, Fuchsberger C, Stephens M, Marchini J, Abecasis GR (2012). Fast and accurate genotype imputation in genome-wide association studies through pre-phasing. Nat. Genet..

[27] Consortium SWGotPG, others. (2014). Biological insights from 108 schizophrenia-associated genetic loci. Nature.

[28] Euesden Jack, Lewis Cathryn M., O’Reilly Paul F. (2014). PRSice: Polygenic Risk Score software. Bioinformatics.

[29] Campbell MC, Tishkoff SA (2008). African genetic diversity: implications for human demographic history, modern human origins, and complex disease mapping. Annu. Rev. Genom. Hum. Genet..

[30] Reed FA, Tishkoff SA (2006). African human diversity, origins and migrations. Curr. Opin. Genet. Dev..

[31] Bryc K, Durand EY, Macpherson JM, Reich D, Mountain JL (2015). The genetic ancestry of African Americans, Latinos, and European Americans across the United States. Am. J. Hum. Genet..

[32] Ha N-T, Freytag S, Bickeboeller H (2014). Coverage and efficiency in current SNP chips. Eur. J. Hum. Genet..

[33] Martin AR (2017). Human demographic history impacts genetic risk prediction across diverse populations. Am. J. Hum. Genet..

[34] Vassos E (2017). An examination of polygenic score risk prediction in individuals with first-episode psychosis. Biol. Psychiatry.

[35] Gandal MJ (2018). Shared molecular neuropathology across major psychiatric disorders parallels polygenic overlap. Science.

[36] Bulik-Sullivan B (2015). An atlas of genetic correlations across human diseases and traits. Nat. Genet..

[37] Wei Z (2013). Large sample size, wide variant spectrum, and advanced machine-learning technique boost risk prediction for inflammatory bowel disease. Am. J. Hum. Genet..

[38] Krapohl E (2016). Phenome-wide analysis of genome-wide polygenic scores. Mol. Psychiatry.

[39] Selzam S (2017). Predicting educational achievement from DNA. Mol. Psychiatry.

[40] Turley Patrick, Walters Raymond K., Maghzian Omeed, Okbay Aysu, Lee James J., Fontana Mark Alan, Nguyen-Viet Tuan Anh, Wedow Robbee, Zacher Meghan, Furlotte Nicholas A., Magnusson Patrik, Oskarsson Sven, Johannesson Magnus, Visscher Peter M., Laibson David, Cesarini David, Neale Benjamin M., Benjamin Daniel J. (2018). Multi-trait analysis of genome-wide association summary statistics using MTAG. Nature Genetics.

[41] Maier RM (2018). Improving genetic prediction by leveraging genetic correlations among human diseases and traits. Nat. Commun..

[42] Krapohl E, Patel H, Newhouse S, Curtis C J, von Stumm S, Dale P S, Zabaneh D, Breen G, O'Reilly P F, Plomin R (2017). Multi-polygenic score approach to trait prediction. Molecular Psychiatry.

[43] Weiner DJ (2017). Polygenic transmission disequilibrium confirms that common and rare variation act additively to create risk for autism spectrum disorders. Nat. Genet..

[44] Tansey KE (2016). Common alleles contribute to schizophrenia in CNV carriers. Mol. Psychiatry.

[45] Levy R. J., Xu B., Gogos J. A., Karayiorgou M. Copy number variation and psychiatric disease risk. Genomic Structural Variants. 97–113 (Springer, 2012). 10.1007/978-1-61779-507-7_4 22228008

[46] Cohen J (2005). Low LDL cholesterol in individuals of African descent resulting from frequent nonsense mutations in PCSK9. Nat. Genet..

[47] Popejoy AB, Fullerton SM (2016). Genomics is failing on diversity. Nature.

[48] Lázaro-Muñoz G, Juengst ET (2015). Challenges for Implementing a PTSD Preventive Genomic Sequencing Program in the US Military. Case W Res J. Int. Law.

[49] Ressler KJ (2011). Post-traumatic stress disorder is associated with PACAP and the PAC1 receptor. Nature.

[50] Guffanti G (2013). Genome-wide association study implicates a novel RNA gene, the lincRNA AC068718.1, as a risk factor for post-traumatic stress disorder in women. Psychoneuroendocrinology.

