# Supplementary material for: Polygenic risk associated with post-traumatic stress disorder onset and severity
Source: Transl Psychiatry. 2019 Jun 7;9:165. doi: 10.1038/s41398-019-0497-3 (PMC6555815; doi:10.1038/s41398-019-0497-3)
Supplement: Supplementary file 1 — Supplementary information - Polygenic risk associated with post-traumatic stress disorder onset and severity [file 41398_2019_497_MOESM1_ESM.docx]

**Supplementary information**

**Polygenic risk associated with post-traumatic stress disorder onset and severity**

# SUPPLEMENTARY METHODS

**Details of the theoretical performance projections**

1. PTSD prevalence and heritability estimates from twin studies

The incidence rate of PTSD varies among different socio-demographic groups. In the U.S. general public, it has a lifetime prevalence rate of about 6.8−8% (9, 10) and up to 15% among OEF/OIF veterans (11, 12). Pioneering PTSD twin studies on Vietnam war veterans, estimated PTSD heritability (defined as the fraction of phenotypic variance attributable to genetic variance) to be about 30% (13); similarly, an heritability between 13% and 34% was shown in symptom clusters (re-experiencing, avoidance, arousal) (14). Comparable levels of heritability was also observed in civilian twin studies (15). Subsequent studies, however, found a higher estimate (about 72%) of heritability for women (16). A study by True, et al. (14) used the largest sample size (2224 monozygotic and 1818 dizygotic male twins) to estimate PTSD heritability and their heritability estimate (30%) is used here for further analysis.

1. Sample size required to explain a given fraction of phenotypic variance

Suppose target phenotype and corresponding predicted scores for $n_{2}$ samples are denoted by $Y_{2}=\left( Y_{12},\ldots, Y_{n_{2}2} \right)^{T}$ and $\hat{S}_{2}=\left( \hat{S}_{1},\ldots,\hat{S}_{n2} \right)^{T}$, respectively. The performance of a genomic profile prediction on a quantitative target phenotype (in our case PTSD severity) is often measured with the square of correlation coefficient between the actual quantitative values and prediction scores, coefficient of determination$R_{\hat{S}_{2},Y_{2}}^{2}=\frac{Cov\left( \hat{S}_{2},Y_{2} \right)^{2}}{Var(\hat{S}_{2})Var(Y_{2})}$. In the case of binary traits, it can be considered as a relationship between the score and a latent continuous variable from which observable binary traits arise (liability threshold model). Under standard assumptions (independence between marker effects and appropriate standardizations), it can be approximated by (17, 18)

$$R_{\hat{S}_{2},Y_{2}}^{2}\approx h^{2}\left( \frac{h^{2}}{h^{2}+\frac{m}{n_{1}}} \right),$$

where $n_{1}$ is the number of samples needed, *m* is the number of markers measured and *h*^2^ is the (narrow sense) heritability on the liability scale of the disease. Obviously, the upper bound on $R^{2}$ is the heritability$h^{2}$, i.e., $\lim_{n\to\infty} R^{2}=h^{2}$.

For cross-disorder prediction, this $R^{2}$ will be attenuated as follows (19)

$$R_{\hat{S}_{2},Y_{2}}^{2}\approx h_{2}^{2}\rho_{G}^{2}\left( \frac{h_{1}^{2}}{h_{1}^{2}+\frac{m}{n_{1}}} \right)$$

where$h_{1}^{2}$is heritability in the base/training phenotype, $h_{2}^{2}$ is heritability in the target phenotype, and $\rho_{G}^{2}$ is genetic correlation between the two phenotypes.

1. Computing the corresponding AUC of an $R^{2}$

For a given pair of samples, one drawn from each class, the probability of the sample from the disease class (cases) having a larger score than the other sample is known as AUC (20). Here it should be noted that, in practice, AUC is usually computed over the ROC curve (TPR vs. FPR plot), hence the name. The corresponding AUC of a given $R^{2}$can be computed as (17, 21)

$$AUC=\Phi\left( \frac{(u-v)R_{\hat{S}_{2},Y_{2}}^{2}}{\sqrt{R_{\hat{S}_{2},Y_{2}}^{2}\left[ 2-R_{\hat{S}_{2},Y_{2}}^{2}u\left( u-T \right)-R_{\hat{S}_{2},Y_{2}}^{2}v(v-T) \right]}} \right)$$

where $\Phi$ denotes the CDF of a standard normal distribution $T=\Phi^{-1}(1-K)$ is the threshold of liability for a disease prevalence rate K, $u=\exp\left( -0.5T^{2} \right)\left( 2\pi K^{2} \right)^{-0.5}$ is the height (ordinate) of the standard normal probability density function (PDF) at the threshold T divided by K, $v=-uK/(1-K)$, and $R_{\hat{S}_{2},Y_{2}}^{2}$ is the coefficient of determination of the panel.

Now, PTSD has a prevalence rate of approximately 8% and a heritability estimate of about 30%. For PTSD, using the above equation, an estimate of the maximum AUC that can be achieved with a solely genetic predictor is approximately 0.8086. And an AUC of 0.75 will be reached when 19.1% of the total variance (or 63.9% of the genetic variation) is explained by the genetic profile, which in turn requires about 300,000 samples, assuming 50,000 contributing markers (which is probably on the higher end (22)) are measured.

Here some caveats need to be noted. PTSD, by definition, is triggered by a traumatic event (Criteria A of DSM-IV), and the level of trauma exposure is not uniform across populations. The 8% prevalence estimate used here is for the general public (comprising those with and without trauma exposure). If conditional PTSD diagnosis probabilities, given trauma exposure, are needed, a higher prevalence rate corresponding to the specific population of interest should be used. For example, prevalence among OEF/OIF veterans is 5−15%, and among those with combat experience is even higher. Another complicating factor includes LD structure between markers (here markers are assumed to be unlinked).

URLs:

GWAS summary statistics data for PTSD and schizophrenia: [https://www.med.unc.edu/pgc/resultsand-downloads](https://www.med.unc.edu/pgc/results-and-downloads)

R statistical computing environment: <https://www.r-project.org/>

PLINK: <https://www.cog-genomics.org/plink2>

PRSice: http://prsice.info/PRSice_v1.25.zip

SHAPEIT: <https://mathgen.stats.ox.ac.uk/genetics> [software/shapeit/shapeit.html](https://mathgen.stats.ox.ac.uk/genetics_software/shapeit/shapeit.html)

Genetic map data: [http://www.shapeit.fr/files/genetic](http://www.shapeit.fr/files/genetic_map_b37.tar.gz) [map](http://www.shapeit.fr/files/genetic_map_b37.tar.gz) [b37.tar.gz](http://www.shapeit.fr/files/genetic_map_b37.tar.gz)

IMPUTE2: [https://mathgen.stats.ox.ac.uk/impute/impute](https://mathgen.stats.ox.ac.uk/impute/impute_v2.html) [v2.html](https://mathgen.stats.ox.ac.uk/impute/impute_v2.html)

Phased reference panel from 1000 Genome Project: [http://www.well.ox.ac.uk/ cfreeman/software/gwas/gtool.h](http://www.well.ox.ac.uk/~cfreeman/software/gwas/gtool.html)

GAS power calculator: [http://csg.sph.umich.edu/abecasis/cats/gas](http://csg.sph.umich.edu/abecasis/cats/gas_power_calculator) [power](http://csg.sph.umich.edu/abecasis/cats/gas_power_calculator) [calculator](http://csg.sph.umich.edu/abecasis/cats/gas_power_calculator)

# SUPPLEMENTARY FIGURES

| 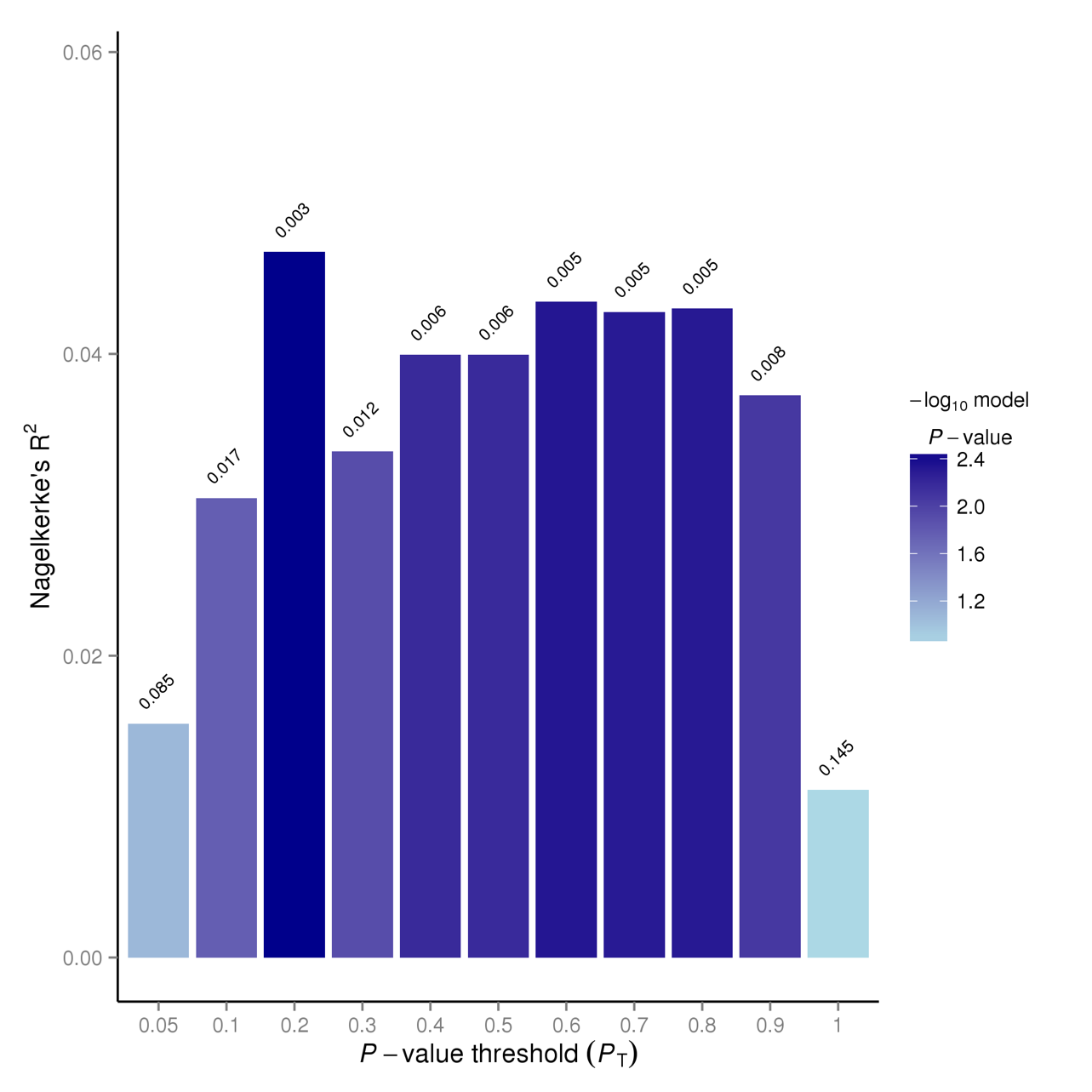 |
| --- |
| Figure S1. P-value thresholding for choosing the optimal threshold after LD clumping. |

|  |  |
| --- | --- |
|  | |
| Figure S2: Ancestral composition of the target (SysBio) cohort. Assigned clusters closely matches self-identified ethnicity/race. Note that even if the illustration is shown for the first two PC’s, the clustering is done on the whole genome-wide data (containing all dimensions of the PC’s). Eigenvalue bar chart showing the fraction of variance explained by the first few principal components. | |

# SUPPLEMENTARY TABLE

Table S1. Genetic clusters, self-identified ethnicity/race of individuals and predictive performance of the PTSD-PRS in the four clusters.

**Supplementary References**

1. Purcell S, Neale B, Todd-Brown K, Thomas L, Ferreira MA, Bender D, et al. PLINK: a tool set for whole-genome association and population-based linkage analyses. Am J Hum Genet. 2007;81(3):559-75.

2. Delaneau O, Marchini J, Zagury J-F. A linear complexity phasing method for thousands of genomes. Nat Methods. 2012;9(2):179.

3. Delaneau O, Marchini J, McVean GA, Donnelly P, Lunter G, Marchini JL, et al. Integrating sequence and array data to create an improved 1000 Genomes Project haplotype reference panel. Nat Commun. 2014;5:3934.

4. Howie B, Fuchsberger C, Stephens M, Marchini J, Abecasis GR. Fast and accurate genotype imputation in genome-wide association studies through pre-phasing. Nat Genet. 2012;44(8):955.

5. Almli LM, Stevens JS, Smith AK, Kilaru V, Meng Q, Flory J, et al. A genome-wide identified risk variant for PTSD is a methylation quantitative trait locus and confers decreased cortical activation to fearful faces. Am J Med Genet Part B. 2015;168(5):327-36.

6. Duncan LE, Ratanatharathorn A, Aiello AE, Almli LM, Amstadter AB, Ashley-Koch AE, et al. Largest GWAS of PTSD (N= 20 070) yields genetic overlap with schizophrenia and sex differences in heritability. Mol Psychiatry. 2017.

7. Consortium SWGotPG, others. Biological insights from 108 schizophrenia-associated genetic loci. Nature. 2014;511(7510):421-7.

8. Purcell SM, Wray NR, Stone JL, Visscher PM, O'donovan MC, Sullivan PF, et al. Common polygenic variation contributes to risk of schizophrenia and bipolar disorder. Nature. 2009;460(7256):748-52.

9. Kessler RC, Berglund P, Demler O, Jin R, Merikangas KR, Walters EE. Lifetime prevalence and age-of-onset distributions of DSM-IV disorders in the National Comorbidity Survey Replication. Arch Gen Psychiatry. 2005;62(6):593-602.

10. Vieweg WVR, Julius DA, Fernandez A, Beatty-Brooks M, Hettema JM, Pandurangi AK. Posttraumatic stress disorder: clinical features, pathophysiology, and treatment. Am J Med. 2006;119(5):383-90.

11. Seal KH, Bertenthal D, Miner CR, Sen S, Marmar C. Bringing the war back home: Mental health disorders among 103 788 US veterans returning from Iraq and Afghanistan seen at Department of Veterans Affairs Facilities. Arch Intern Med. 2007;167(5):476-82.

12. Ramchand R, Karney BR, Osilla KC, Burns RM, Caldarone LB. Prevalence of PTSD, depression, and TBI among returning servicemembers. In: Tanielian T, Jaycox LH, editors. Invisible Wounds of War: Psychological and Cognitive Injuries, Their Consequences, and Services to Assist Recovery: RAND Corporation; 2008. p. 35-86.

13. Lyons MJ, Goldberg J, Eisen SA, True W, Tsuang MT, Meyer JM, et al. Do genes influence exposure to trauma?: A twin study of combat. Am J Med Genet. 1993;48(1):22-7.

14. True WR, Rice J, Eisen SA, Heath AC, Goldberg J, Lyons MJ, et al. A twin study of genetic and environmental contributions to liability for posttraumatic stress symptoms. Arch Gen Psychiatry. 1993;50(4):257-64.

15. Stein MB, Jang KL, Taylor S, Vernon PA, Livesley WJ. Genetic and environmental influences on trauma exposure and posttraumatic stress disorder symptoms: a twin study. Am J Psychiatry. 2002;159(10):1675-81.

16. Sartor CE, McCutcheon VV, Pommer NE, Nelson EC, Grant JD, Duncan Ae, et al. Common genetic and environmental contributions to post-traumatic stress disorder and alcohol dependence in young women. Psychol Med. 2011;41(07):1497-505.

17. Daetwyler HD, Villanueva B, Woolliams JA. Accuracy of predicting the genetic risk of disease using a genome-wide approach. PLoS One. 2008;3(10):3395.

18. Dudbridge F. Power and predictive accuracy of polygenic risk scores. PLos Genet. 2013;9(3):1003348.

19. De Vlaming R, Okbay A, Rietveld CA, Johannesson M, Magnusson PK, Uitterlinden AG, et al. Meta-GWAS Accuracy and Power (MetaGAP) calculator shows that hiding heritability is partially due to imperfect genetic correlations across studies. PLos Genet. 2017;13(1):1006495.

20. Hanley JA, McNeil BJ. The meaning and use of the area under a receiver operating characteristic (ROC) curve. Radiology. 1982;143(1):29-36.

21. Wray NR, Yang J, Goddard ME, Visscher PM. The genetic interpretation of area under the ROC curve in genomic profiling. PLos Genet. 2010;6(2):1000864.

22. Zhang Y, Qi G, Park J-H, Chatterjee N. Estimation of complex effect-size distributions using summary-level statistics from genome-wide association studies across 32 complex traits and implications for the future. bioRxiv. 2017:175406.
